# Inosine-Containing dsRNA Binds a Stress-Granule-like Complex and Downregulates Gene Expression In *trans*

**DOI:** 10.1016/j.molcel.2007.09.005

**Published:** 2007-11-09

**Authors:** A.D.J. Scadden

**Affiliations:** 1 Department of Biochemistry, University of Cambridge, Cambridge CB2 1GA, UK

**Keywords:** RNA

## Abstract

Long double-stranded RNAs (dsRNAs) may undergo extensive modification (hyperediting) by adenosine deaminases that act on RNA (ADARs), where up to 50% of adenosine (A) residues are changed to inosine (I). Traditionally, consequences of A-to-I editing were thought to be limited to modified RNA itself. We show here, however, that hyperedited dsRNA (I-dsRNA) is able to downregulate gene expression in *trans*. Furthermore, we show that both endogenous expression and reporter gene expression were substantially reduced in the presence of I-dsRNA. This was due to a reduction in reporter mRNA levels and also translation inhibition. Importantly, we show that I-dsRNA interferes with translation initiation. We also show that I-dsRNA specifically binds a stress-granule-like complex. Stress granules (SGs) are important for translational silencing during stress. Finally, we propose a model whereby editing by ADARs results in downregulation of gene expression via SG formation.

## Introduction

Long double-stranded RNA (dsRNA) in cells is often indicative of viral infection (Maquat and Carmichael, 2001). Alternatively, it may result from expression of both sense and antisense RNAs, or from the presence of inverted repeat sequences within noncoding RNA. Cells respond to long dsRNA molecules by activating general antiviral systems mediated by enzymes such as PKR (Samuel, 1998). Alternatively, dsRNA may undergo covalent modification (editing) by adenosine deaminases that act on RNA (ADARs) (Bass, 2002), or be used in the RNAi pathway (Filipowicz et al., 2005).

ADARs catalyze the hydrolytic deamination of adenosine (A) to inosine (I) (Bass, 2002). As I is decoded as guanosine (G) by ribosomes, selective editing has the potential to alter the coding capacity of mRNAs. ADARs also catalyze hyperediting within relatively long dsRNAs, where up to 50% of A residues are converted to I. Hyperediting changes not only the RNA sequence but also the structure, as IU and UI pairs have different geometry than AU and UA pairs (Serra et al., 2004). Localized distortions within the RNA helix are likely to result from the presence of IU pairs.

The majority of mammalian editing occurs within noncoding regions of RNA, such as inverted repeat sequences within intronic or intergenic RNAs, or within untranslated regions (UTRs). The most frequent targets of editing are high copy number repetitive elements, such as Alus (Blow et al., 2004; Levanon et al., 2004; Morse et al., 2002). It has therefore been predicted that >85% of pre-mRNAs may be edited. A-to-I editing has also been found within miRNA precursors, which has the potential to affect both miRNA production and also target recognition (Blow et al., 2006; Kawahara et al., 2007; Yang et al., 2006).

Hyperedited dsRNAs (I-dsRNAs) in cells may be subject to different fates. I-dsRNA may be retained in the nucleus by a protein complex comprising p54^nrb^, PSF, and matrin 3 (Zhang and Carmichael, 2001). Alternatively, hyperediting by ADARs could provide a means of covalently “tagging” dsRNA for subsequent disposal. We have previously identified a nuclease activity in various cytoplasmic extracts that specifically targets I-dsRNA (Scadden and Smith, 2001). Cleavage occurred within sequences containing multiple IU pairs, but not in duplexes that contained either isosteric GU pairs or Watson-Crick base pairs. Tudor staphylococcal nuclease (TSN), which is a component of the RNA-induced silencing complex in the RNAi pathway (Filipowicz et al., 2005), is important for cleavage of I-dsRNA (Scadden, 2005). Cleavage of I-dsRNA may play a role in viral defense or in destroying cellular dsRNAs edited by ADARs.

During stress, eukaryotic cells have the ability to reprogram their ribosomes to selectively synthesize proteins needed for survival (Anderson and Kedersha, 2006). As part of this process, a subset of cellular mRNAs are translationally silenced by sequestration into cytoplasmic stress granules (SGs). As the mechanism underlying SG assembly appears to be impaired translation initiation (Anderson and Kedersha, 2006), SGs comprise stalled initiation complexes and small ribosomal proteins (Kedersha et al., 2002). When stress conditions are relieved, sequestered mRNAs may reassemble on polysomes to resume translation (Kedersha et al., 2000). Alternatively, mRNAs may be destroyed in cytoplasmic processing bodies (P bodies), which are dynamically linked to SGs (Kedersha et al., 2005). SGs therefore comprise sites of triage where mRNAs are sorted for storage, reinitiation, or degradation.

Here we demonstrate that I-dsRNA specifically binds a complex that comprises proteins previously characterized as SG components. Furthermore, we show that I-dsRNA in cells results in downregulating both endogenous and reporter gene expression, in *trans*. We provide evidence that this is the result of both reduced mRNA levels and impaired translation. Moreover, we show that I-dsRNA inhibits translation initiation. We therefore propose a model in which hyperediting of dsRNAs by ADARs results in downregulation of gene expression via SG formation.

## Results

### I-dsRNA Binding Proteins

**Figure 1:**
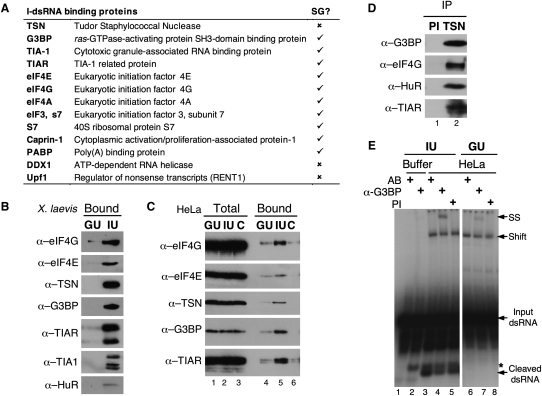
A “Stress-Protein Complex” Forms Specifically on I-dsRNA (A) Proteins that bind specifically to I-dsRNA. Previous characterization as an SG component is indicated. (B) An immunoblot comprising *X. laevis* proteins eluted from GU and IU affinity matrices was probed with various antibodies. (C) Immunoblots were used to analyze proteins bound to GU, IU, and C dsRNAs in HeLa lysates (lanes 4–6). Total protein is also shown (lanes 1–3). (D) α-TSN IPs from HeLa lysates were analyzed by immunoblotting with antibodies against SG proteins. Preimmune serum (PI) was used as a control. (E) When IU dsRNA was incubated with HeLa lysate, an RNA-protein complex was detected using EMSA (Shift; lanes 3–5). Cleaved I-dsRNA and a nonspecific cleavage product (^∗^) were also observed. A super-shifted (SS) complex was seen when α-G3BP was added (lane 4), but not with antibody buffer (AB) or PI (lanes 3 and 5, respectively). A GU RNA-protein complex also formed in HeLa lysate (lanes 6–8), and addition of α-G3BP resulted in a super-shifted complex (lane 7). This was absent with either AB or PI (lanes 6 and 8, respectively).

dsRNA affinity matrices were previously used to identify specific I-dsRNA binding proteins, such as TSN (Scadden, 2005). We have now used a similar approach to identify additional I-dsRNA binding proteins. Briefly, *X. laevis* oocyte extract was incubated with a GU dsRNA affinity matrix to deplete nonspecific proteins, then with either a specific I-dsRNA (IU; Table 1) affinity matrix or a second GU dsRNA matrix (see Figure S1 in the Supplemental Data available with this article online). 2D-Dige was used to analyze proteins eluted from the final GU and IU matrices (Figure S1B), and I-dsRNA binding proteins were subsequently identified. Interestingly, most of these proteins were SG components (Figure 1A and Table S1). Where antibodies were available, immunoblots were used to confirm that the *X. laevis* proteins identified did bind specifically to I-dsRNA (Figure 1B). Immunoblotting was also used to demonstrate the presence of an additional SG component, HuR, that was not identified by protein sequencing (Anderson and Kedersha, 2006).

We next asked whether the specificity of binding of the *X. laevis* proteins to I-dsRNA was maintained in HeLa cell lysates. Specific (IU) or nonspecific (GU, C) dsRNAs were therefore used for HeLa cell affinity purification. Immunoblots were subsequently used to demonstrate that the I-dsRNA stress-complex proteins identified in *X. laevis* also bound preferentially to I-dsRNA in HeLa cell lysates (Figure 1C, lanes 4–6). Furthermore, immunoprecipitations (IP) using a TSN antibody in HeLa lysates showed that some of the proteins were found in an RNA-independent complex (Figure 1D). It was not possible to look at all possible proteins due to a lack of suitable antibodies.

Formation of an HeLa protein complex on I-dsRNA could additionally be shown using electrophoretic mobility shift assays (EMSA) (Figure 1E). When IU dsRNA was incubated with HeLa lysate, an RNA-protein complex with retarded mobility (“Shift”) was observed (lanes 3–5). Moreover, a band with increased mobility that corresponds to cleavage of I-dsRNA (Scadden, 2005) was also present (lanes 3–5). A similar shifted RNA-protein complex was also seen when GU dsRNA was incubated with HeLa lysate (lanes 6–8). This was in contrast to *X. laevis* oocyte extract where RNA-protein complex formation using GU dsRNA was undetectable (Scadden, 2005). Minor differences in the composition of the complex in *X. laevis* and HeLa may account for the observed difference in binding. Nevertheless, complex formation on GU dsRNA was less efficient than with IU dsRNA, as the IU dsRNA-protein complex was rapidly turned over due to cleavage (Scadden, 2005). To confirm that the SG protein G3BP was present in the dsRNA-protein complex, a G3BP antibody was added to the assay with either IU or GU dsRNA. This gave rise to a “super-shifted” (SS) complex (lanes 4 and 7) that did not form when either antibody buffer (AB) or preimmune serum (PI) were added (lanes 3 and 6 and lanes 5 and 8, respectively). A similar analysis was used to confirm the presence of TSN in the dsRNA-protein complex (Figure S2). These data provided independent evidence that both G3BP and TSN were components of the I-dsRNA complex.

### I-dsRNA Reduced Expression of Luciferase Reporters

As the I-dsRNA complex comprised SG proteins, we speculated that the presence of I-dsRNA in cells could induce SG formation, which would result in downregulating mRNA expression. To test this hypothesis, reporter plasmids and various synthetic dsRNAs (Table 1) were used to cotransfect HeLa cells. Short dsRNAs were used to avoid activation of PKR. Transfection conditions favored efficient uptake of the dsRNAs (>95% of cells), as judged by visualization of fluorescently labeled duplexes (data not shown).

**Figure 2:**
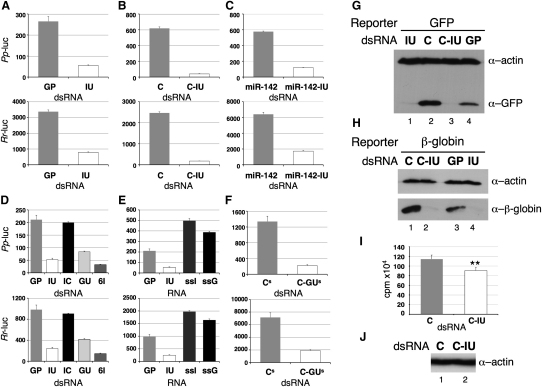
I-dsRNA Reduced the Expression of Various Reporters *Pp*-luc and *Rr*-luc activity was measured following expression in HeLa cells. All error bars are mean ± SD, n = 3. (A–C) *Pp*-luc and *Rr*-luc were expressed in the presence of GP and IU ([A], n > 10), C and C-IU ([B], n > 20), and miR-142 and miR-142-IU ([C], n = 6) dsRNAs. (D) *Pp*-luc and *Rr*-luc were expressed with GP, IU, IC, GU, and 6I dsRNAs (n = 4). (E) *Pp*-luc and *Rr*-luc were expressed in the presence of GP and IU dsRNAs, and ssI and ssG (n = 5). (F) Expression of *Pp*-luc and *Rr*-luc was analyzed with C-GU^s^ and C^s^ dsRNAs (n = 3). (G and H) Immunoblotting was used to analyze GFP (G) and β-globin (H) expression in the presence of C and C-IU, and GP and IU dsRNAs. Actin was used as a loading control. (I) HeLa cells were labeled with [^35^S]Met following transfection with C or C-IU, and total protein synthesis was determined by TCA precipitation (n = 10). A t test gave p = 1.9 × 10^−5^ (^∗∗^). Error bars are mean ± SD, n = 10. (J) An immunoblot of the labeled cell lysates was probed with α-actin as a loading control.

The first reporters tested were firefly (*Pp*-luc) and renilla luciferase (*Rr*-luc), together with the GP or IU dsRNAs (Table 1). The GP and IU duplexes were identical except for the four central base pairs; the GP control dsRNA consisted of perfect Watson-Crick base pairs, while the IU dsRNA contained IU pairs. The *Pp*-luc and *Rr*-luc reporters shared no sequence homology with each other, or with the dsRNAs. The reporter plasmids were cotransfected with either IU or GP into HeLa cells, and luciferase (luc) assays were carried out after 30 hr. In the presence of IU dsRNA, expression of both *Pp*-luc and *Rr*-luc was substantially reduced relative to that in the presence of GP (Figure 2A). This observation was also true when cells were harvested after 12–72 hr, and when the amount of specific dsRNA added was reduced 4-fold (data not shown). To ensure that the observed effect was unrelated to the sequence of the dsRNA per se, a second pair of dsRNAs, C and C-IU, were tested in the assay using luc reporters (Table 1). Again, cotransfection of C-IU I-dsRNA with both luc reporters caused a substantial reduction in their expression relative to that with the control (C) dsRNA (Figure 2B). Finally, a third pair of dsRNAs whose sequence corresponded to a miRNA duplex were tested for their effect on expression of the luc reporters. It was previously shown that the miR-142 pri-miRNA precursor was edited by ADARs both in vitro and in vivo (Yang et al., 2006). Editing gave rise to numerous I residues in the sequences corresponding to both sense and antisense miRNAs, although the level of mature miRNAs was reduced due to inefficient processing (Yang et al., 2006). Nevertheless, a pair of dsRNAs that mimicked either unedited miR-142 (miR-142) or edited miR-142 (miR-142-IU) were tested (Table 1). Again, in the presence of the I-dsRNA, miR-142-IU, expression of *Pp*-luc and *Rr*-luc was reduced relative to that with unedited miR-142 (Figure 2C).

These data demonstrated conclusively that I-dsRNA was able to downregulate gene expression in a sequence-independent manner. Of particular interest was the observation that miR-142-IU caused reduced gene expression, which suggests that edited miRNAs have the potential for regulating gene expression in *trans*.

### IU Pairs Are Necessary for Reduced Expression

To test whether IU pairs were required to reduce reporter expression or if I was sufficient per se, the IC dsRNA (Table 1) was tested in parallel with IU and GP, in conjunction with the luc reporters. IC differed from IU in that it contained IC pairs in the central region. When luc assays were performed following transfection of the various duplexes, only the IU duplex reduced *Pp*-luc and *Rr*-luc expression (Figure 2D). IU pairs were therefore necessary for inhibiting reporter expression. The GU duplex, which contains isosteric GU pairs rather than IU pairs (Table 1), was also tested. Cotransfection of GU dsRNA with the luc reporters resulted in a substantial reduction in their expression, relative to that with GP, although it was ∼2-fold less potent than the IU duplex (Figure 2D). This observation was consistent with differential complex formation on IU and GU dsRNAs (Figure 1). It is possible that the distortion in the RNA helix resulting from inclusion of either IU or GU pairs is important for recognition of proteins, ultimately resulting in reduced gene expression. When a duplex containing six IU pairs (6I; Table 1) was cotransfected with the luc reporters, it was more effective than IU dsRNA at inhibiting expression (Figure 2D). Conversely, duplexes that contained only two IU pairs were ineffective at reducing reporter expression (data not shown). I-containing ssRNA was also unable to inhibit gene expression (Figure 2E). These data suggested that only dsRNAs containing multiple IU or GU pairs were able to efficiently inhibit gene expression.

### Expression of GU Duplexes In Vivo Reduced Gene Expression

We next asked whether dsRNAs transcribed within the cell rather than by transfection were also able to reduce reporter gene expression. Plasmids based on pSuper, which was designed for use in RNAi, were therefore constructed in order to produce dsRNAs equivalent to either C or C-IU in vivo. However, as both GU and IU dsRNAs inhibited luc expression (Figure 2D), GU pairs were substituted for IU pairs in the C-IU duplex. The pSuper-C and pSuper-C-GU constructs gave rise to C^S^ and C-GU^S^ dsRNAs, respectively (Table 1). As seen with the transfected dsRNAs, C-GU^S^ dsRNA resulted in substantially reduced expression of both luc reporters, relative to that with C^S^ (Figure 2F). These data confirmed that gene expression was reduced by dsRNAs containing multiple IU or GU pairs, irrespective of how they were introduced into cells.

### I-dsRNA Reduced Expression of GFP and β-Globin Reporters

The expression of two additional reporters (GFP and β-globin) was next examined, in conjunction with two pairs of dsRNAs (C and C-IU, GP and IU). Again, the reporters and dsRNAs were unrelated. Gene expression was analyzed by immunoblotting. With the C and C-IU duplexes, expression of both GFP and β-globin was substantially reduced in the presence of C-IU relative to C dsRNA (Figure 2G, lanes 2 and 3, and Figure 2H, lanes 1 and 2, respectively). Similarly, expression of both GFP and β-globin was reduced in the presence of IU dsRNA compared with GP (Figure 2G, lanes 1 and 4, and Figure 2H, lanes 3 and 4, respectively). An actin antibody was used as a loading control. As a large pool of presynthesized actin exists in HeLa cells, we assumed the levels would be unaffected by I-dsRNA. These data again demonstrate that I-dsRNA had a substantial effect on gene expression, in a sequence-independent manner.

### I-dsRNA Reduced Endogenous Protein Synthesis

We have shown conclusively that I-dsRNAs reduced expression of various reporter genes. We next asked whether I-dsRNA in cells also downregulated endogenous protein synthesis.

To analyze the effect of I-dsRNA on endogenous protein synthesis, HeLa cells were transfected with either C or C-IU dsRNAs, then metabolically labeled with [^35^S]methionine ([^35^S]Met) 24 hr posttransfection. In the presence of the C-IU dsRNA, 20%–25% less [^35^S]Met was incorporated into newly synthesized endogenous proteins, relative to that seen with the control (C) duplex (Figure 2I). An immunoblot of the cell lysates was probed with α-actin as a loading control (Figure 2J). Analysis of the ^35^S-labeled proteins by SDS-PAGE followed by phosphorimaging suggested that all detectable protein bands were less abundant (data not shown). These data clearly demonstrated that I-dsRNA was able to downregulate endogenous gene expression.

### I-dsRNAs Reduce mRNA Levels

I-dsRNA caused a substantial reduction in expression of four different reporters, relative to control dsRNAs. We subsequently carried out experiments to see whether I-dsRNA affected reporter mRNA levels.

**Figure 3:**
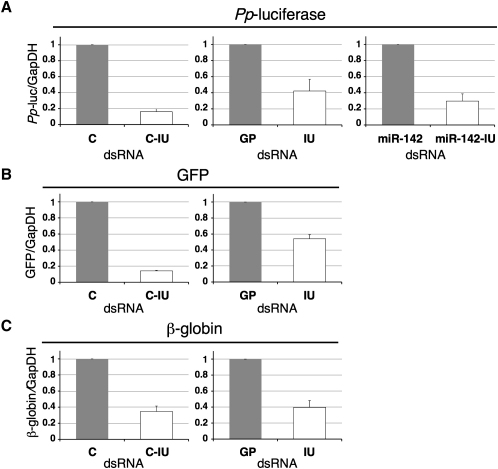
mRNA Levels Were Reduced by I-dsRNA RT/qPCR was used to quantify reporter mRNAs. Fold change in mRNA levels in the presence of I-dsRNA was calculated relative to the control dsRNA, and normalized to GAPDH (n ≥ 4). Error bars are mean ± SD, n ≥ 4. (A) *Pp*-luc mRNA was measured in the presence of I-dsRNAs (C-IU, IU, or miR-142-IU), relative to control dsRNAs (C, GP, or miR-142, respectively). GFP (B) and β-globin (C) mRNA levels were also determined with I-dsRNA (C-IU or IU) relative to control dsRNAs (C or GP, respectively).

Reverse transcription (RT) and quantitative PCR (qPCR) were initially used to analyze *Pp*-luc mRNA levels in experiments in which luc reporters were cotransfected with three different pairs of dsRNAs (C and C-IU, GP and IU, and miR-142 and miR-142-IU). The amount of mRNA in the presence of I-dsRNA was calculated relative to that seen with the non-I-dsRNA control, and normalized to GAPDH. For each pair of dsRNAs, the reporter mRNA was reduced in the presence of I-dsRNA (C-IU, IU, miR-142-IU, Figure 3A). RNA from transfections with the GFP reporter and two pairs of dsRNAs (C and C-IU, GP and IU) was next analyzed. Again, the GFP mRNA was substantially reduced with C-IU and IU, relative to the control dsRNAs (Figure 3B). The final reporter analyzed was β-globin mRNA, which differed from the other reporters in that it required splicing for subsequent expression. This was verified using standard RT-PCR (data not shown). RT/qPCR was again used to show that in the presence of two different I-dsRNAs, C-IU and IU, β-globin mRNA levels were reduced relative to those seen with either C or GP duplexes, respectively (Figure 3C). These data together showed that I-dsRNA in cells resulted in a substantial reduction in reporter mRNA levels. Nuclear run on analyses suggested that this occurred posttranscriptionally (data not shown).

### I-dsRNAs Cause Translational Inhibition

While I-dsRNA caused a reduction in the level of reporter mRNAs (Figure 3), this did not appear sufficient to wholly account for the substantial decrease in reporter gene expression (Figures 2A–2H). It was possible to estimate, at least for the luc reporters, that the reduction in gene expression in the presence of I-dsRNA was at least 2-fold greater than the fold-reduction in reporter mRNA levels. In keeping with the idea that I-dsRNA reduced gene expression via SG assembly, we therefore asked whether translation was also inhibited by I-dsRNA.

**Figure 4:**
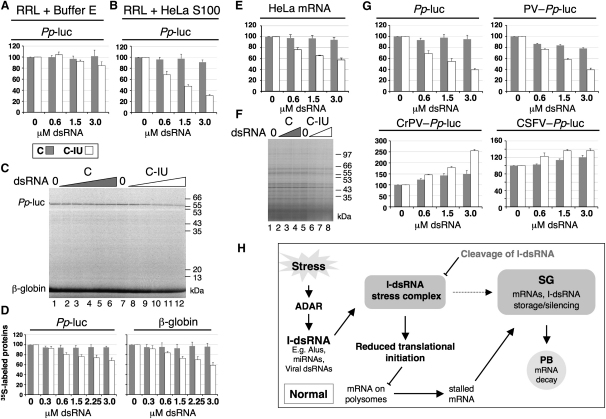
I-dsRNA Inhibits Translation Initiation In Vitro (A) *Pp*-luc mRNA was translated using RRL/buffer E, with increasing amounts of C or C-IU dsRNAs (n = 6). Luc assays were used to quantify translation efficiency, and the *Pp*-luc activity was expressed as a percentage relative to that seen in the absence of dsRNA. Error bars are mean ± SD, n ≥ 3. (B) *Pp*-luc mRNA was translated in RRL/S100, with increasing amounts of C and C-IU, and quantified as in (A) (n > 12). (C) *Pp*-luc mRNA was translated using non-MN-treated RRL/S100 with increasing amounts of C (lanes 1–6) or C-IU (lanes 7–12). The addition of [^35^S]Met enabled analysis by SDS-PAGE. (D) Protein bands corresponding to *Pp*-luc or globin (in [C]) were quantified (n = 4). The amount of translated product is given as a percentage of that observed in the absence of dsRNA. All error bars are mean ± SD, n = 4. (E) Total HeLa mRNA was translated using RRL/S100, with increasing concentrations of C and C-IU dsRNAs (n = 6). Translation was quantified by TCA precipitation of the ^35^S-labeled proteins, and the amount of translated product is shown as a percentage of that in the absence of dsRNA. (F) ^35^S-labeled proteins generated by translation of total HeLa mRNA in the presence of C (lanes 1–4) or C-IU (lanes 5–8) were separated by SDS-PAGE. (G) *Pp*-luc and IRES-*Pp*-luc mRNAs were translated using RRL/S100, with increasing amounts of C or C-IU dsRNAs (n ≥ 3). Translation was quantified as in (A). (H) A model for how I-dsRNA reduces gene expression via SG formation.

To address this question, we analyzed translation of *Pp*-luc reporter mRNA in vitro using micrococcal nuclease (MN)-treated rabbit reticulocyte lysate (RRL), in the presence of C or C-IU dsRNAs. Luc assays were subsequently used to quantify translation efficiency, and the *Pp*-luc activity was expressed as a percentage of the luc activity in the absence of dsRNA. In translation reactions using RRL alone, increasing amounts of C and C-IU dsRNAs had no effect on translation of *Pp*-luc mRNA (Figure 4A). However, when the RRL was supplemented with cytoplasmic HeLa extract (S100), translation of *Pp*-luc mRNA was reduced with increasing amounts of C-IU dsRNA (Figure 4B). In contrast, addition of C dsRNA had a negligible effect on translation. It is possible that the requirement for S100 was due to the absence of TSN in RRL (Figure S3A). This idea was supported by the observation that translation of *Pp*-luc mRNA in RRL supplemented with recombinant TSN (rTSN) was reduced in the presence of C-IU relative to that seen with C dsRNA (Figure S3B). While rTSN was less effective than S100 in reducing expression of *Pp*-luc mRNA, this may be due to inefficient recruitment of rTSN to protein complexes in RRL, or perhaps because another protein component was also absent.

The translation inhibition observed with increasing concentrations of C-IU dsRNA was not the result of decreased stability of *Pp*-luc mRNA, as determined by RT/qPCR (Figure S4). These data demonstrated that I-dsRNA caused translational inhibition in vitro.

### I-dsRNA Reduced Translation of Endogenous mRNAs

I-dsRNA inhibited translation of exogenous *Pp*-luc mRNA in vitro. We next asked whether translation of endogenous mRNAs in vitro was also reduced in the presence of C-IU dsRNA.

We initially analyzed the effect of C-IU on translation of endogenous RRL globin mRNA. Translation reactions were therefore assembled using non-MN-treated RRL/S100, and [^35^S]Met was added to enable visualization of the translation products. In the presence of increasing concentrations of C-IU dsRNA, a corresponding decrease in translation of both *Pp*-luc and globin mRNA was seen (Figure 4C, lanes 7–12, Figure 4D). Although the reduction of *Pp*-luc translation appeared less than observed previously (Figure 4B), this was likely to be due to competition with other mRNAs in the non-MN-treated RRL. As expected, no decrease in translation was observed with increasing amounts of C dsRNA, for either *Pp*-luc or globin mRNAs (Figure 4C, lanes 1–6, and Figure 4D).

Translation of total HeLa mRNA was then analyzed using MN-treated RRL/S100, where [^35^S]Met was added to enable quantification. In the presence of increasing amounts of C-IU dsRNA, a corresponding decrease in translation of HeLa mRNA was observed, relative to that seen with C dsRNA (Figure 4E). ^35^S-labeled proteins generated by in vitro translation of HeLa mRNA were also visualized by SDS-PAGE. A decrease in the intensity of all detectable protein bands was seen with increasing amounts of C-IU dsRNA, relative to the control duplex (Figure 4F, lanes 5–8 and lanes 1–4, respectively).

These data showed that I-dsRNA had an inhibitory effect on protein synthesis in vitro and were consistent with the observation that I-dsRNA resulted in downregulating protein synthesis in vivo (Figure 2I).

### I-dsRNA Inhibited Initiation of Translation

We have shown that I-dsRNA inhibited translation of many mRNAs. We therefore considered whether I-dsRNA interfered with either translation initiation or elongation.

To investigate the effect of I-dsRNA on translation initiation, several reporter mRNAs were used for translation that differed in their requirement for initiation factors. These reporters depended on viral internal ribosome entry sites (IRESs) for recognition of the start codon, rather than cap-dependent ribosome scanning. The additional reporters used were poliovirus-*Pp*-luc (PV-*Pp*-luc), classical swine fever virus-*Pp*-luc (CSFV-*Pp*-luc), and cricket paralysis virus-*Pp*-luc (CrPV-*Pp*-luc). PV-*Pp*-luc requires all canonical factors except eIF4E; CSFV-*Pp*-luc does not require eIF4E, eIF4G, eIF4A, eIF4B, eIF1, or eIF1A; and CrPV-*Pp*-luc requires no canonical initiation factors. All of the luc reporters were capped and polyadenylated and were translated using MN-treated RRL/S100. The translation efficiency was measured by luc assays, as described above.

As expected, addition of increasing amounts of C-IU dsRNA resulted in a corresponding decrease in translation of *Pp*-luc mRNA, while C dsRNA had no effect (Figure 4G). For PV-*Pp*-luc mRNA, translation was also inhibited with increasing amounts of C-IU dsRNA, relative to translation with C dsRNA. In contrast, the C-IU dsRNA did not inhibit translation of either CrPV-*Pp*-luc or CSFV-*Pp*-luc mRNAs. Moreover, translation was increased at higher concentrations of C-IU dsRNA relative to that with an equivalent amount of C dsRNA. This observation was particularly evident for the CrPV-*Pp*-luc mRNA. It is possible that at high C-IU concentrations sufficient initiation factors were titrated out by I-dsRNA complex formation (Figure 1) so that CrPV-*Pp*-luc, which requires no canonical initiation factors, has a competitive advantage for translation. Equivalent results were obtained when the *Pp*-Luc or IRES-*Pp*-luc mRNAs, along with C or C-IU dsRNAs, were injected into *X. laevis* oocytes (data not shown). These data unequivocally demonstrate that I-dsRNA inhibits translation initiation. We therefore concluded that I-dsRNA caused a reduction in reporter gene expression by reducing mRNA levels and by interfering with translation initiation.

## Discussion

To the best of our knowledge, the data presented here provide insights that suggest new roles for I-dsRNA. While the effects of A-to-I editing were previously thought to be restricted to the modified RNA itself, we now see that I-dsRNA is able to inhibit gene expression in *trans* (Figures 2–4). We have therefore proposed a model to account for our observations (Figure 4H). Briefly, our model predicts that I-dsRNA is generated during stress as the result of editing by ADARs. I-dsRNA binds the “stress-complex,” which in turn induces gene silencing via SG assembly. The rationale for the proposed model is discussed here.

During cellular stress, up to 50% of mRNAs may be translationally silenced by sequestration in SGs (Anderson and Kedersha, 2006). Transiently transfected reporters may also be silenced by induction of SGs (Kedersha et al., 2000). Our findings that both endogenous protein synthesis and reporter gene expression are reduced in the presence of I-dsRNA (Figure 2) are therefore consistent with SG formation and selective silencing of mRNAs under stress conditions.

A key determinant for SG assembly is impaired translation initiation (Anderson and Kedersha, 2006). In view of the fact that I-dsRNA inhibits gene expression by interfering with translation initiation (Figure 4), this is a plausible route by which I-dsRNA could induce SG assembly. Translation initiation is likely to be blocked by I-dsRNA due to sequestration of initiation factors in the I-dsRNA complex (Figure 1A). It is also feasible that the I-dsRNA complex is sufficient to nucleate SG assembly, facilitated by proteins such as TIA-1 and G3BP (Anderson and Kedersha, 2006). However, the data presented here favor the idea that I-dsRNA could give rise to SGs as the result of impaired translation initiation.

I-dsRNA not only inhibits translation initiation but also results in reduced levels of reporter mRNAs (Figure 3). As there is a close connection between mRNA processing events in SGs and P bodies (Kedersha et al., 2005), we predict that mRNAs are degraded in P bodies. Moreover, a previous study showed that a transiently expressed GFP reporter gene was localized to both SGs and P bodies (Kedersha et al., 2005). A combination of both translational silencing and mRNA degradation therefore appears to be responsible for reduced gene expression observed in the presence of I-dsRNA.

We have used model dsRNAs to show that I-dsRNA and GU dsRNA can downregulate gene expression (Figures 2 and 4). It is therefore important to consider where such dsRNAs would occur naturally. While GU pairs commonly occur within RNA duplexes, they are generally only found in tandem (Masquida and Westhof, 2000). By analogy with I-dsRNA, it is unlikely that two GU pairs are sufficient to downregulate gene expression. I-dsRNA is produced by A-to-I editing by ADARs (Bass, 2002). As discussed above, common editing substrates include noncoding repetitive elements such as Alus, as well as miRNA precursors (Blow et al., 2004, 2006; Levanon et al., 2004). dsRNA arising from viral infection may also be edited by cytoplasmic ADAR1. While the I-dsRNAs used in this study were relatively short compared to naturally occurring substrates, they constitute an effective model for edited dsRNA. It is therefore expected that cellular I-dsRNAs would also have the potential to inhibit gene expression. Regulation may be achieved by differential expression of editing targets during adverse conditions. In the case of viral infection, dsRNA would only be present during infection. Alternatively, ADAR itself may be regulated during stress.

Cytoplasmic ADAR1 is induced by interferon (George and Samuel, 1999), which supports the idea that it is important for viral defense, and also in response to serum starvation (Wang et al., 2004). Moreover, *ADAR1^−/−^* mice were unviable, with extensive apoptosis in embryonic tissues normally expressing high levels of ADAR1 (Wang et al., 2004). Apoptosis similarly occurred in *ADAR1^−/−^* MEFs during serum starvation. Editing of an unknown substrate by ADAR1 was thought to be necessary for protection against stress-induced apoptosis. It is possible that I-dsRNA-induced SG assembly may facilitate cell survival during stress conditions. dADAR mutant flies had an increased resistance to reactive oxygen species (ROS) (Chen et al., 2004). This was the result of selective changes in gene expression, which included upregulation of two genes encoding potential ROS scavengers. Conversely, these genes were downregulated when dADAR was overexpressed. Changes in expression appeared to be indirectly regulated by dADAR. This is consistent with our model whereby editing results in reduced gene expression in *trans*.

TSN interacts with and promotes specific cleavage of I-dsRNA (Scadden, 2005). We previously speculated that cleavage was important as part of an antiviral mechanism or to regulate aberrantly edited cellular dsRNAs. As TSN is an integral part of the I-dsRNA stress complex (Figure 1D), we now propose that cleavage of I-dsRNA could provide an efficient means of switching off the I-dsRNA-induced silencing pathway. It is intriguing that TSN is activated in mouse vascular smooth muscle cells during oxidative stress (Sakamoto et al., 1999).

We have put forward a provocative model whereby gene regulation occurs in response to A-to-I editing by ADARs. In accordance with this model, I-dsRNA has the potential to alter localization, stability or translation of mRNA, in *trans*. However, we cannot rule out the possibility that the observations made using I-dsRNA represent a more general quality control pathway in the cell that could be activated by other aberrantly structured RNAs. Future work will shed light on the scope of the proposed pathway.

## Experimental Procedures

### Transfections

HeLa cells (2 × 10^5^ cells/well) were transfected using Lipofectamine-2000 (Invitrogen). For cotransfection of dsRNA and DNA, 120 pmol dsRNA (80 pmol specific dsRNA + 40 pmol C dsRNA), and 0.2–1 μg DNA were used. Luciferase, 0.19 μg pGL3-con + 0.01 μg pRL-SV40 (Promega); GFP, 0.2 μg pEGFP-C1 (BD Biosciences); β-globin, 1 μg β-globin (βΔ5–7) + 0.2 μg Tat (Dye et al., 2006). For pSuper-C-GU and pSuper-C, cells were transfected with 1 μg DNA, then after 48 hr with luc reporters. Cells were harvested with TRI reagent (Sigma) for RNA, or passive lysis buffer (PLB; Promega) for luc assays. For immunoblots or IPs, cells were lysed in RIPA or NET buffer on ice for 25 min, and spun at 10,000 rpm for 10 min (4°C).

### Luciferase Assays

Equal amounts of protein (determined by Bradford assays) were assayed using the Dual-Luciferase Reporter Assay System (Promega). Data shown are representative of multiple experiments (where all transfections were in triplicate) for each pair of dsRNAs, as indicated in figure legends.

### In Vitro Translation

Assays comprised 45% RRL, 100 mM KCl, 0.5 mM MgCl_2_, 10 mM CP, 100 μM amino acids, and 25%–30% (v/v) HeLa S100 (or buffer E) (Scadden and Smith, 2001). *Pp*-luc mRNA (3 nM) and 0.5 μl of C or C-IU dsRNA (0–3 μM) were added. Where indicated, 50 ng HeLa mRNA, prepared using oligo(dT)25 dynabeads, was used. Reactions were incubated at 30°C for 90 min. [^35^S]Met (0.5 μl) was added for analysis by SDS-PAGE or TCA precipitation. Data were quantified using a Molecular Dynamics phosphorimager and Imagequant software.

### RT/qPCR

DNase-treated RNA (2–5 μg) was used for RT using AMV-RT (Promega) and oligo dT. –RT controls were carried out. qPCR was done with a Rotor-Gene 6000 (Corbett Research) using SYBR green (Bioline). Fold change in mRNA levels was calculated relative to the control and normalized to GAPDH. See Supplemental Data for primers.

### dsRNA Affinity Matrices

Affinity matrices comprised biotinylated dsRNAs (Dharmacon) linked to magnetic streptavidin beads (Dynal) and were incubated with lysates as described previously (Scadden, 2005). Proteins were eluted with ASB14 lysis buffer (8 M urea, 2% ASB14, protease inhibitors) and analyzed by 2D-DiGE. CyDye labeling, 2D protein separation, gel imaging, and analysis were performed as described previously (Karp et al., 2004). I-dsRNA binding proteins were identified by mass spectrometry (Scadden, 2005).

### HeLa dsRNA Affinity Purification

HeLa cells were transfected with biotinylated dsRNAs. Cell lysates were prepared after 30 hr, and RNA complexes were recovered by incubation on ice with magnetic streptavidin beads. Beads were washed extensively with cold 10 mM HEPES-KOH/100 mM NaCl buffer, and proteins were eluted with SDS buffer.

### Metabolic Labeling

HeLa cells were transfected using C or C-IU dsRNAs, then labeled 24 hr later with [^35^S]Met. For labeling, cells were incubated in Met-free media for 30 min, then [^35^S]Met (∼3 MBq/ml) was added for a further 15 min. Cell lysates were prepared in RIPA buffer, and [^35^S]Met incorporation was quantified by TCA precipitation.

## Figures and Tables

**Table 1:** dsRNA Substrates

Substrate	Sequence																										
C					G	G	U	C	C	G	G	C	**U**	**C**	**C**	**C**	C	C	A	A	A	U	G	d	T	d	T
	d	T	d	T	C	C	A	G	G	C	C	G	**A**	**G**	**G**	**G**	G	G	U	U	U	A	C				
C-IU					G	G	U	C	C	G	G	C	**I**	**I**	**U**	**I**	C	C	A	A	A	U	G	d	T	d	T
	d	T	d	T	C	C	A	G	G	C	C	G	**U**	**U**	**I**	**U**	G	G	U	U	U	A	C				

GP					A	C	U	G	G	A	C	A	**G**	**G**	**U**	**G**	C	U	C	C	G	A	G	G			
					U	G	A	C	C	U	G	U	**C**	**C**	**A**	**C**	G	A	G	G	C	U	C	C			
IU					A	C	U	G	G	A	C	A	**I**	**I**	**U**	**I**	C	U	C	C	G	A	G	G			
					U	G	A	C	C	U	G	U	**U**	**U**	**I**	**U**	G	A	G	G	C	U	C	C			
IC					A	C	U	G	G	A	C	A	**I**	**I**	**U**	**I**	C	U	C	C	G	A	G	G			
					U	G	A	C	C	U	G	U	**C**	**C**	**A**	**C**	G	A	G	G	C	U	C	C			
GU					A	C	U	G	G	A	C	A	**G**	**G**	**U**	**G**	C	U	C	C	G	A	G	G			
					U	G	A	C	C	U	G	U	**U**	**U**	**G**	**U**	G	A	G	G	C	U	C	C			
6I					C	U	G	G	A	C	A	**I**	**I**	**U**	**I**	**U**	**U**	C	U	C	C	G	A	G			
					G	A	C	C	U	G	U	**U**	**U**	**I**	**U**	**I**	**I**	G	A	G	G	C	U	C			
ssI					A	C	U	G	G	A	C	A	**I**	**I**	**U**	**I**	C	U	C	C	G	A	G	G			
ssG					A	C	U	G	G	A	C	A	**G**	**G**	**U**	**G**	C	U	C	C	G	A	G	G			

miR-142															A												
					C	A	**U**	**A**	**A**	**A**	G	**U**	**A**	G		**A**	A	G	C	A	C	**U**	**A**	C			
				G	G	U	**A**	**U**	**U**	**U**	C	**A**	**U**	C		**U**	U	U	G	U	G	**A**	**U**	G	U		
															C												
miR-142-IU															A												
					C	A	**U**	**I**	**I**	**I**	G	**U**	**I**	G		**I**	A	G	C	A	C	**U**	**I**	C			
				G	G	U	**I**	**U**	**U**	**U**	C	**I**	**U**	C		**U**	U	U	G	U	G	**I**	**U**	G	U		
															C												

C^s^					G	G	U	C	C	G	G	C	**U**	**C**	**C**	**C**	C	C	A	A	A	U	G	U	U		
			U	U	C	C	A	G	G	C	C	G	**A**	**G**	**G**	**G**	G	G	U	U	U	A	C				
C-GU^s^					G	G	U	C	C	G	G	C	**G**	**G**	**U**	**G**	C	C	A	A	A	U	G	U	U		
			U	U	C	C	A	G	G	C	C	G	**U**	**U**	**G**	**U**	G	G	U	U	U	A	C				

## Supplemental Data

Document S1. Supplemental Experimental Procedures, Supplemental References, Four Figures, and One Table
