# Supplementary material for: Inosine-Containing dsRNA Binds a Stress-Granule-like Complex and Downregulates Gene Expression In trans
Source: Mol Cell. 2007 Nov 9;28(3):491–500. doi: 10.1016/j.molcel.2007.09.005 (PMC2075533; doi:10.1016/j.molcel.2007.09.005)
Supplement: Document S1. Supplemental Experimental Procedures, Supplemental References, Four Figures, and One Table [file mmc1.pdf]

Molecular Cell, Volume *28*

Supplemental Data

Inosine-Containing dsRNA Binds a Stress-Granule-like Complex and Downregulates Gene Expression In *trans*

A.D.J. Scadden

Experimental Procedures

RNAs

dsRNAs were prepared according to manufacturers instructions (Dharmacon). Capped mRNAs were prepared by *in vitro* transcription (Scadden and Smith, 2001). For *Pp*-luc mRNA, pLuc-MCS (Wormington et al., 1996) was linearized with *Pvu*II. PV-*Pp*-luc, CrPV-*Pp*-luc, CSFV-*Pp*-luc (Gorgoni et al., 2005) constructs were linearized with *Bgl*II. RNAs were polyadenylated *in vitro* (Ambion).

Plasmids

pSuper-C (C^S) was constructed by cloning pre-annealed C^s sense and antisense oligonucleotides (see below) into the *Bgl*II and *Hin*dIII sites of pSuper (Oligoengine). Similarly, pSuper-C-GU (C-GU^S) was constructed by cloning C-GU^s sense and antisense oligonucleotides.

Immunoblots

Proteins were transferred to Immobilon P following separation by SDS-PAGE. Membranes were blocked for >1 h in TBST containing 2% (w/v) milk powder. Primary antibodies used were: α-GFP (Invitrogen), α-TSN (ADJS), α-G3BP and α-

eIF4G (BDBiosciences), α-TIAR, α-TIA-1, α-HuR and α-Hemoglobin-β (Santa Cruz Biotechnology), α-eIF4G and α-eIF4E (gifts from Simon Morley). Secondary antibodies were horseradish peroxidase coupled (Jackson ImmunoResearch). Visualization was by enhanced chemiluminescence.

Immunoprecipitations (IPs)

10 μl of beads (Protein A Sepharose coupled to polyclonal α-TSN or PI serum) were mixed with 50 μl RNase A treated HeLa lysate in 1 ml NET buffer, and incubated with rotation for 2 h at 4 °C. Beads were washed extensively with NET buffer and proteins eluted using SDS sample buffer.

EMSA

HeLa NET lysates (20% (v/v)) were used, as described previously (Scadden, 2005). Antisera were affinity purified (Protein A Sepharose). 'AB' was equivalent to the antibody buffer.

Oligonucleotides

C^s sense:

5'GATCCCCGGTCCGGCTCCCCCAAATGTTCAAGAGACATTTGGGGGAGCC GGACCTTTTTGGAAA

C^s antisense:

5'AGCTTTTCCAAAAAGGTCCGGCTCCCCCAAATGTCTCTTGAACATTTGG GGGAGCCGGACCGG

C-GU^s sense:

5'GATCCCCGGTCCGGCGGTGCCAAATGTTCAAGAGACATTTGGTGTTGCC GGACCTTTTTGGAAA

C-GU^{s} antisense:

5’AGCTTTTCCAAAAAGGTCCGGCGGTGCCAAATGTCTCTTGAACATTTGG TGTTGCCGGACCGGG

Primer Pairs Used for qPCR

GFP:

Forward: 5’-CAGGAGCGCACCATCTTCTT

Reverse: 5’-CGATGCCCTTCAGCTCGAT

Pp-luc:

Forward: 5’-TCGCCAGTCAAGTAACAAC

Reverse: 5’-ACTTCGTCCACAAACACAA

Human β-globin:

Forward: 5’-TCCACTCCTGATGCTGTTA

Reverse: 5’-AGGCACCGAGCACTTTCT

Rabbit globin:

Forward: 5’-TCCTCTGCAAATGCTGTTA

Reverse: 5’-CTTGCCATGAGCCTTCAC

GapDH:

Forward: 5’-TGCACCACCACCTGCTTAGC

Reverse: 5’-GGCATGGACTGTGGTCATGAG.

In Vitro Translation with rTSN

In vitro translation was carried out essentially as described in experimental procedures. However, when 5 pmol rTSN (Scadden, 2005) was added to translations reactions, HeLa S100 was omitted. Translations with rTSN (or a control with HeLa S100) were carried out in the presence of 1.5 µM C or C-IU dsRNA. Luciferase activity is shown as a ratio of that seen with C-IU dsRNA relative to C dsRNA.

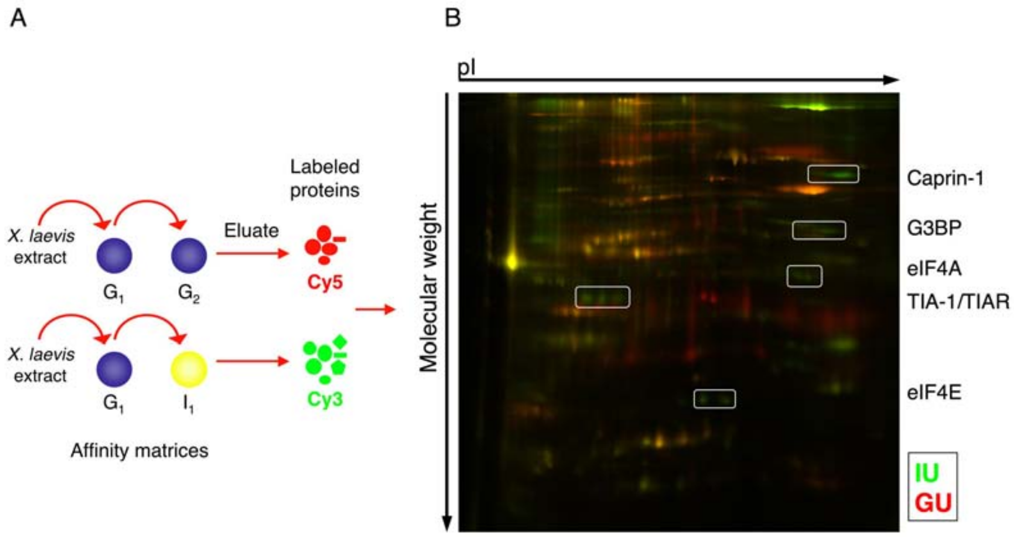

Figure S1. dsRNA Affinity Matrices Were Used to Identify I-dsRNA Binding Proteins

(A) A schematic representation of the tandem affinity matrices used to purify I-dsRNA binding proteins from *X. laevis* oocyte extract.

(B) Proteins eluted from GU and IU affinity matrices were analyzed by 2D-difference gel electrophoresis (2D-DiGE). Proteins bound specifically to the I-dsRNA affinity matrix appeared as green spots. Proteins were subsequently identified; some examples are shown.

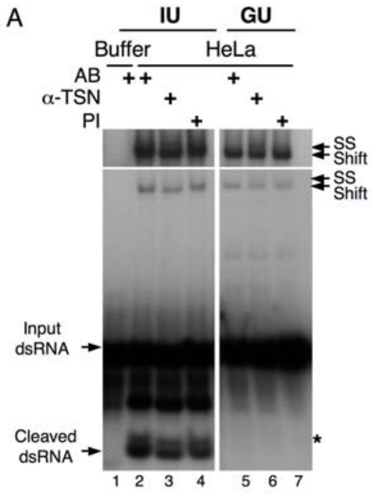

Figure S2. A "Stress-Protein Complex" Forms Specifically on I-dsRNA

(A) An RNA-protein complex was seen using EMSA when both IU and GU dsRNAs were incubated in HeLa cell lysate (lanes 2–4 and 5–7, respectively). This was not seen with buffer alone (lanes 1). While a dsRNA-protein complex formed with both IU and GU dsRNA, formation was less efficient with GU dsRNA. In addition to the RNA-protein complex, cleaved I-dsRNA was also readily detectable (lanes 2–4). A non-specific cleavage product was also seen (*). Addition of α-TSN resulted in a weak super-shifted species for both IU and GU dsRNAs (compare lanes 3 and 6, respectively, with lanes 2, 4, 5 and 7), whose mobility was only slightly less than the shifted band. A longer exposure of the top part of the gel is shown. As the super-shift was relatively difficult to visualize, the presence of TSN in the complex was additionally verified by EMSA using a HeLa extract where TSN was depleted using RNAi (data not shown).

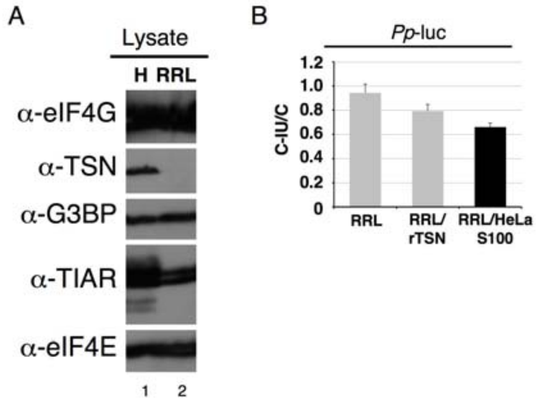

Figure S3. TSN Is Required for Translation Inhibition by I-dsRNA

(A) An immunoblot of HeLa S100 ('H') and RRL was probed with various antibodies against SG components.

(B) *Pp*-luc mRNA was translated using RRL alone or RRL supplemented with either rTSN or HeLa S100, in the presence of 1.5 μM C or C-IU dsRNA (n=6). Translation was subsequently measured using luciferase assays, and was expressed as a ratio of luciferase activity in the presence of C-IU relative to C. All error bars are mean ± SD, n ≥ 3. A Student's *t*-test (2-tails, unequal variance) gave a P-value of <0.005.

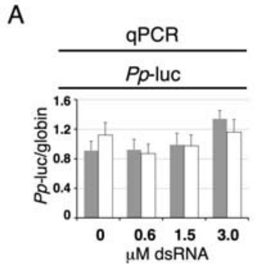

Figure S4. mRNA Was Stable during In Vitro Translation with I-dsRNA

(A) RT-qPCR was used to analyze stability of *Pp*-luc mRNA during *in vitro* translation. Fold-change in mRNA levels was normalized to endogenous rabbit globin mRNA. All error bars are mean ± SD, n ≥ 3.

Table S1. Proteins that Bind Specifically to I-dsRNA

Protein		Peptides	SG? (reference)
**TSN**	Tudor Staphylococcal Nuclease	16	×
**G3BP**	*ras*-GTPase-activating protein SH3-domain binding protein	18	✓(Tourriere et al., 2003)
**TIA-1**	Cytotoxic granule-associated RNA binding protein	9	✓(Kedersha et al., 1999)
**TIAR**	TIA-1 related protein	2	✓(Kedersha et al., 1999)
**eIF4E**	Eukaryotic initiation factor 4E	5	✓(Kedersha et al., 2002)
**eIF4G**	Eukaryotic initiation factor 4G	11	✓(Kedersha et al., 2002)
**eIF4A**	Eukaryotic initiation factor 4A	5	✓(Low et al., 2005)
**eIF3, s7**	Eukaryotic initiation factor 3, subunit 7	5	✓(Kedersha et al., 2002)
**S7**	40S ribosomal protein S7	2	✓(Kedersha et al., 2002)
**Caprin-1**	Cytoplasmic activation/proliferation-associated protein 1	15	✓(Solomon et al., 2007)
**PABP**	Poly(A) binding protein	2	✓(Kedersha et al., 1999)
**DDX1**	Human ATP-dependent RNA helicase	4	×
**Upf1**	Regulator of nonsense transcripts (RENT1)	4	×

The I-dsRNA binding proteins identified are shown, and the number of unique peptides obtained for each protein is given. Previous characterization as a SG component is indicated for each protein.

Supplemental References

Kedersha, N., Chen, S., Gilks, N., Li, W., Miller, I.J., Stahl, J., and Anderson, P. (2002). Evidence that ternary complex (eIF2-GTP-tRNA(i)(Met))-deficient preinitiation complexes are core constituents of mammalian stress granules. Mol. Biol. Cell *13*, 195-210.

Kedersha, N.L., Gupta, M., Li, W., Miller, I., and Anderson, P. (1999). RNA-binding Proteins TIA-1 and TIAR Link the Phosphorylation of eIF-2a to the Assembly of Mammalian Stress Granules. J. Cell Biol. *147*, 1431-1442.

Low, W.K., Dang, Y.J., Schneider-Poetsch, T., Shi, Z.G., Choi, N.S., Merrick, W.C., Romo, D., and Liu, J.O. (2005). Inhibition of eukaryotic translation initiation by the marine natural product pateamine A. Mol. Cell *20*, 709-722.

Scadden, A.D.J. (2005). The RISC subunit Tudor-SN binds to hyper-edited double-stranded RNA and promotes its cleavage. Nat. Struct. Mol. Biol. *12*, 489-496.

Solomon, S., Xu, Y.X., Wang, B., David, M.D., Schubert, P., Kennedy, D., and Schrader, J.W. (2007). Distinct structural features of caprin-1 mediate its interaction with G3BP-1 and its induction of phosphorylation of eukaryotic translation initiation factor 2 alpha, entry to cytoplasmic stress granules, and selective interaction with a subset of mRNAs. Mol. Cell Biol. *27*, 2324-2342.

Tourriere, H., Chebli, K., Zekri, L., Courselaud, B., Blanchard, J.M., Bertrand, E., and Tazi, J. (2003). The RasGAP-associated endoribonuclease G3BP assembles stress granules. J. Cell Biol. *160*, 823-831.
